# Optical Scattering Evolution during Ambient Aging of Cs/FA Alloyed Perovskite Thin Films

**DOI:** 10.1021/acsomega.6c01763

**Published:** 2026-05-30

**Authors:** Lucas Caniati Escaliante, Lucas Jorge Affonço, Stevan Brayan Oliveira dos Santos, Larissa de Oliveira Garcia, Silvia Leticia Fernandes, André Luis de Jesus Pereira, Carlos Frederico de Oliveira Graeff, José Humberto Dias da Silva

**Affiliations:** † School of Sciences, Graduate Program in Materials Science and Technology—POSMAT, Universidade Estadual Paulista—UNESP, Bauru, São Paulo 17033-360, Brazil; ‡ School of Sciences, Physics and Meteorology Department, Universidade Estadual Paulista—UNESP, Bauru, São Paulo 17033-360, Brazil; § ONINN Centro de Inovação, Belo Horizonte 31035-536, Minas Gerais, Brazil; ∥ Faculty of Physical Engineering/Computer Sciences, 39013 University of Applied Sciences Zwickau, Zwickau 08056, Germany; ⊥ Plasma and Processes Laboratory, Division of Fundamental Sciences, Instituto de Tecnologia Aeronáutica—ITA, São José dos Campos, São Paulo 12228-900, Brazil

## Abstract

This work investigates the optical stability of formamidinium–cesium lead halide perovskite thin films deposited on fluorine-doped tin oxide substrates and aged under ambient conditions for 21 days. The optical response was analyzed through specular and diffuse transmittance and reflectance measurements, collected with light incident from both sides of the heterostructure. Specular transmittance exhibits nonmonotonic variations with an initial increase followed by a gradual decrease over time, while diffuse transmittance increases systematically across the full spectral range, indicating the progressive formation of scattering centers. Total reflectance decreases monotonically with aging, revealing that degradation is primarily governed by absorption-related optical losses. Despite these changes, the absorption edge remains stable, and the optical bandgap and Urbach tail show no significant variation. Direction-dependent measurements demonstrate that the fluorine-doped tin oxide substrate is the dominant source of initial scattering whereas the perovskite layer initially reduces optical contrast and later introduces disorder as degradation progresses. Haze values remain nearly constant over time, indicating that changes in scattering efficiency are moderate compared to absorption losses. These results demonstrate that integrating sphere-based optical spectroscopy provides a nondestructive and effective framework for monitoring early stage degradation in perovskite thin films.

## Graphical abstract

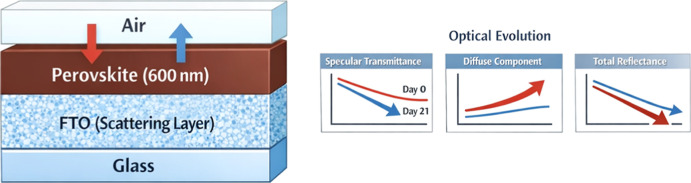

## Introduction

Solar cells based on lead halide perovskites (PSCs) have attracted significant attention due to their remarkable power conversion efficiencies, which have already exceeded 25% in laboratory-scale devices.^1,2^ In addition, when incorporated into tandem architectures with absorbers such as silicon or Cu­(In_1–*x*_Ga_*x*_)­Se_2_ (CIGS), these materials demonstrate outstanding performance, enabling efficiencies that surpass those of single-junction devices fabricated under comparable conditions.^3,4^

Mixed-cation mixed-halide perovskites such as FA_0.83_Cs_0.17_Pb­(Br_0.17_I_0.83_)_3_ have emerged as key materials for high-performance optoelectronic applications, particularly in perovskite solar cells and tandem architectures, due to their improved phase stability, tunable bandgap, and enhanced resistance to environmental degradation compared to conventional MA-based (methylammonium-based) perovskites.^5–9^ These properties make them highly relevant for studying degradation mechanisms under realistic conditions.

Despite these advances, the widespread implementation of this class of solar cells is hindered by their limited environmental stability and short operational lifetimes.^10,11^ The perovskite layer undergoes substantial degradation upon exposure to humidity, oxygen, thermal stress, and continuous illumination, thereby reducing its optical absorption and overall device performance.^12^ Consequently, enhancing the intrinsic stability of the perovskite absorber remains a critical challenge in the field.

A promising strategy to enhance device durability involves the incorporation of dopants that partially replace organic cations and inorganic ions within the perovskite lattice.^13–15^ Such substitutions can suppress degradation pathways by stabilizing the crystal structure and reducing defect-mediated decomposition. In particular, the incorporation of Cs^+^ and Br^–^ into the FAPbI_3_ lattice has been shown not only to slow degradation under light, temperature, and humidity stress but also to support high photovoltaic performance.^16^ Devices based on this alloyed composition, when integrated into tandem architectures with other perovskites or with silicon, can achieve efficiencies exceeding 25%.^13^ These combined improvements in stability and efficiency are key reasons why Cs- and Br-alloyed FAPbI_3_ are used in perovskite solar cells. Among the compositions reported in the literature,^17^ the hybrid perovskite FA_0.83_Cs_0.17_Pb­(Br_0.17_I_0.83_)_3_, a mixed-cation mixed-halide alloyed perovskite in which Cs^+^ partially substitutes FA^+^ at the A-site and Br^–^ partially replaces I^–^ at the X-site of the ABX_3_ lattice, commonly abbreviated as Cs_17_/Br_17_, combines enhanced stability with high photovoltaic efficiency. For these reasons, it was selected as the material of interest in the present work.

The selected composition (FA_0_._83_Cs_0_._17_) falls within the commonly reported range of mixed-cation perovskites, where Cs incorporation is widely used to improve phase stability and reduce sensitivity to environmental degradation.^18–21^ The exact Cs fraction (0.17) does not represent a uniquely optimized value but rather lies within the established compositional window known to enhance structural stability compared to MA-based perovskites.^18–21^

Understanding the origins of instability in these devices, however, remains challenging, particularly when exploring the bulk interfaces without damaging the film. Perovskites exhibit a highly mixed ionic–electronic behavior, where mobile ions, vacancies, and charged defects can redistribute under illumination, bias, or temperature.^22^ These processes occur predominantly at buried interfaces that are not accessible by most surface-sensitive tools. Although techniques such as X-ray Photoelectron Spectroscopy (XPS), Scanning Electron Microscopy (SEM), and Atomic Force Microscopy (AFM) provide valuable information about the near-surface region, they offer limited penetration depth and often require complex sample preparation. In contrast, optical spectroscopies, being simpler, noninvasive, and straightforward to implement, can probe not only the surface but also deeper regions within the bulk, allowing access to features that are otherwise difficult to resolve.

Optical measurements, such as specular and diffuse transmittance and reflectance, provide a nondestructive way to examine thin-film heterostructures. Because the light penetration depth varies with the wavelength, these measurements capture information not only from the surface but also from deeper regions of the film. This makes it possible to detect changes related to disorder, scattering, and other signs of degradation, offering a practical method for monitoring the overall stability of the material.

In this context, the present work aims to investigate the optical evolution of perovskite thin films during aging by combining specular and diffuse measurements. Unlike conventional approaches that focus primarily on absorption, this study highlights the critical role of scattering and structural disorder in governing the optical response, which is often overlooked in previous studies.^5–7^

By analyzing the transmittance and reflectance of light upon incidence from different sides of the sample, this approach enables the identification of the regions that contribute most significantly to scattering and disorder as well as their temporal evolution. This provides a more comprehensive and physically consistent description of the degradation processes in perovskite thin films.

By tracking the temporal evolution of the optical response, this work offers insights into degradation mechanisms while establishing a practical and effective framework for evaluating the long-term stability of mixed-cation perovskite films.

Most previous studies have primarily focused on absorption-related changes to evaluate the degradation in perovskite films. However, recent works have shown that structural disorder and morphological inhomogeneities can significantly affect light scattering and overall optical response. In this context, the present work differs from conventional approaches by explicitly considering both specular and diffuse optical components, providing a more comprehensive description of the degradation mechanisms.

## Results and Discussion

The perovskite film thickness was estimated to be approximately 600 nm, based on previous measurements reported by our research group for films prepared under identical deposition conditions.^23^ While the thickness was not directly measured for the present samples, the reproducibility of the fabrication protocol allows for reliable estimation within this range.

In Figure 1a, the specular transmittance was measured for both the bare substrate and the perovskite sample with illumination from both sides (glass side and perovskite side). No significant differences are observed when comparing the FTO/glass and glass/FTO measurements, in both the interference fringes and the absorption edge regions overlap, showing that the optical response is independent of the illumination direction. In contrast, when the perovskite layer is incorporated, the glass/FTO/PVK incidence exhibits a slight reduction in transmittance between 800 and 1400 nm when compared to the measurements performed in the inverted incidence direction (PVK/FTO/glass). This difference reflects the additional absorption and scattering introduced by the perovskite layer as well as the asymmetry of the heterostructure when the incident beam interacts first with the perovskite film or the glass/FTO substrate. The same trend is observed in the diffuse reflectance curves shown in Figure 2b, although the differences are less pronounced for the samples containing the perovskite layer. In this case, the directional asymmetry of the optical response is still present, but the contribution of the perovskite film to diffuse scattering is comparatively weaker than that in the specular transmittance measurements.

**Figure 1:**
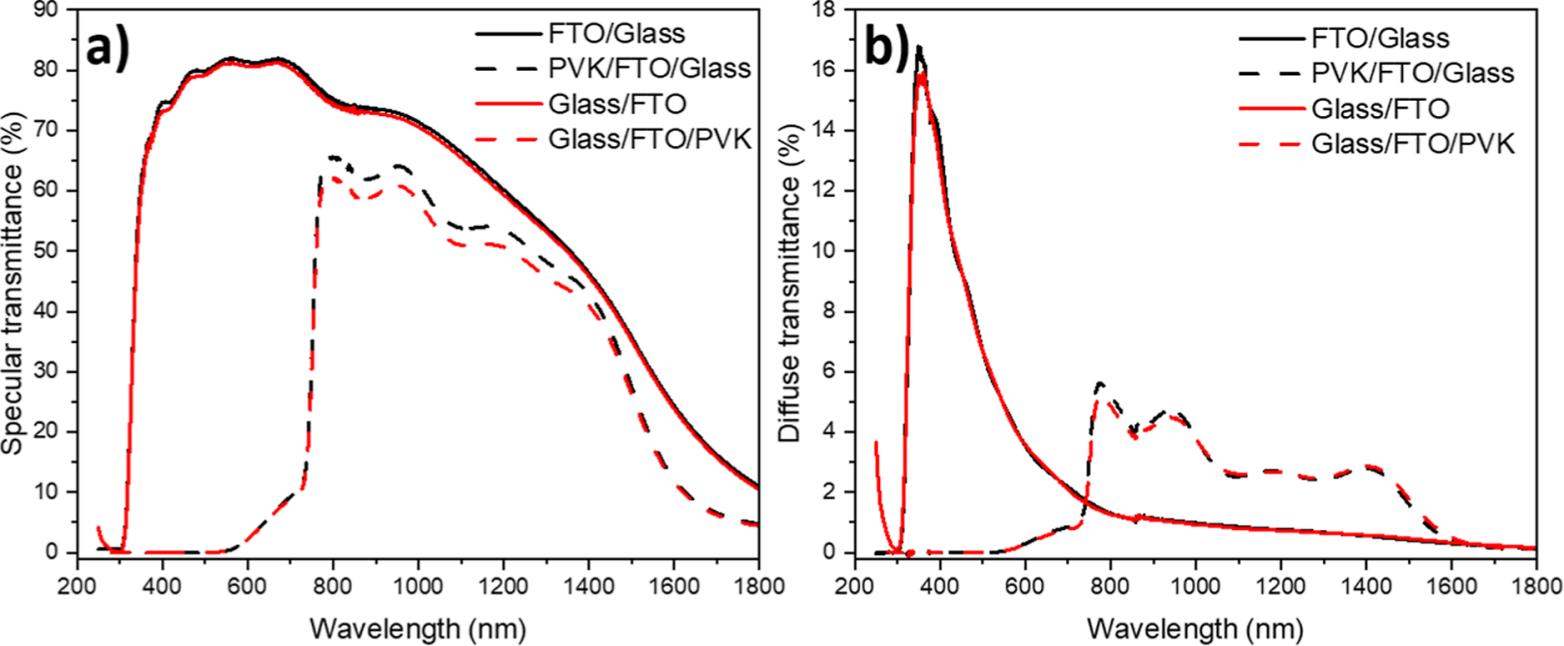
(a) Specular and (b) diffuse transmittance spectra of the FTO (glass/FTO) substrate and the pristine PVK film (glass/FTO/PVK), with light incidence from the film side to glass side.

**Figure 2:**
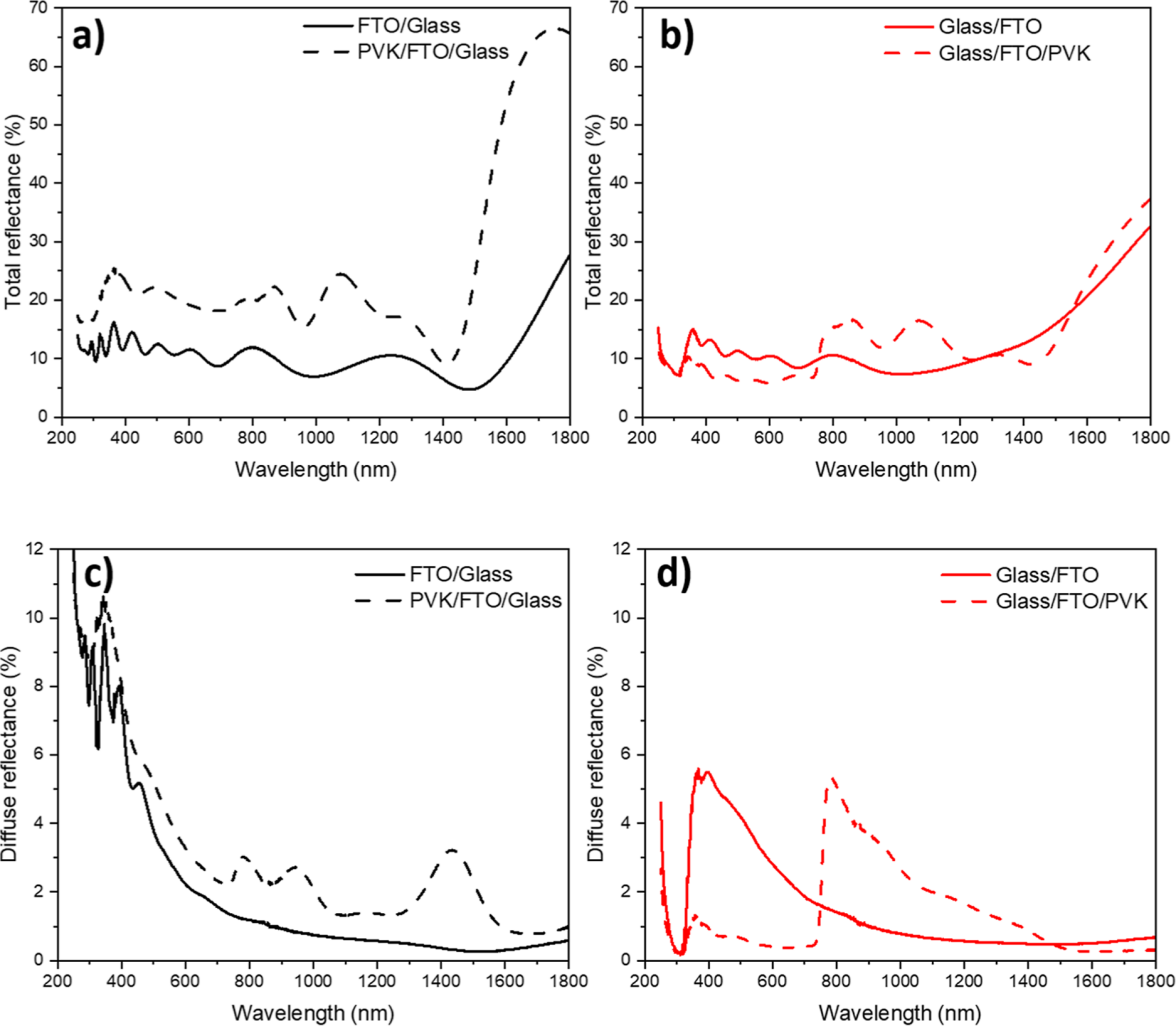
Total reflectance in (a) and (b), and the diffuse reflectance in (c) and (d). (a) Being the measurements performed by the film side and (b,d) the measurements performed by the glass side. The PVK film is in the as-grown condition.

The comparison between the total reflectance curves in Figure 2a (FTO/Glass vs PVK/FTO/Glass) and Figure 2b (Glass/FTO vs Glass/FTO/PVK) reveals noteworthy differences depending on the sample orientation when the PVK layer is present. In Figure 2a, the PVK/FTO/Glass configuration consistently shows higher total reflectance than the FTO/Glass reference. This behavior can be attributed to the larger refractive index contrast at the air/PVK interface, which increases the Fresnel specular reflectance. In contrast, in Figure 2b, where illumination occurs in the Glass/FTO/PVK direction, a higher reflectance than the reference Glass/FTO sample shows up in specific spectral regions (e.g., 800–1200 nm and 1600–1800 nm). In this geometry, the incident light first encounters the air/glass interface, which has a lower refractive index contrast, resulting in reduced Fresnel reflection and a partial suppression of scattering contributions from the underlying FTO layer. These differences highlight the dependence of the optical response on the direction of incidence and on the sequence of interfaces within the heterostructure.

The diffuse reflectance behavior (Figure 2c,d) further highlights the role of interface sequence and optical contrast. The glass/FTO substrate already exhibits significant diffuse reflectance, confirming that the FTO layer is an intrinsic source of scattering due to its morphology. Upon deposition of the perovskite film, the diffuse component is modulated by the combined effects of refractive index matching at the FTO/PVK interface and possible morphological irregularities within the perovskite layer. Consequently, the observed diffuse reflectance arises from the interplay between intrinsic FTO roughness and additional scattering contributions introduced by the perovskite film rather than from a single dominant interface. Overall, these results demonstrate that the optical response of the heterostructure is strongly dependent on the direction of incidence and the sequence of interfaces encountered by the incident light.

### Analysis over Time

The following figures summarize the temporal evolution of the optical response of the PVK/FTO/glass samples stored under ambient conditions.

In Figure 3a, the specular transmittance shows an overall change relative to Day 0, but the evolution does not follow a specific trend. The spectral shape and the position of the absorption edge remain essentially unchanged, indicating that the fundamental optical transitions of the perovskite are preserved during aging. However, instead of a continuous upward shift, the transmittance rises during the first days, reaches a maximum around Day 7–8, and then gradually decreases toward Day 21. This initial increase may be associated with a delayed crystallization or structural relaxation of residual intermediate phases formed during the perovskite deposition process, leading to a temporary reduction in optical losses. At later stages, the subsequent decrease in transmittance suggests the onset of additional scattering or absorption losses related to degradation processes. While more complex light–matter interaction mechanisms, such as electronic light scattering, cannot be completely excluded, the observed behavior is consistently explained by increased structural disorder and scattering.

**Figure 3:**
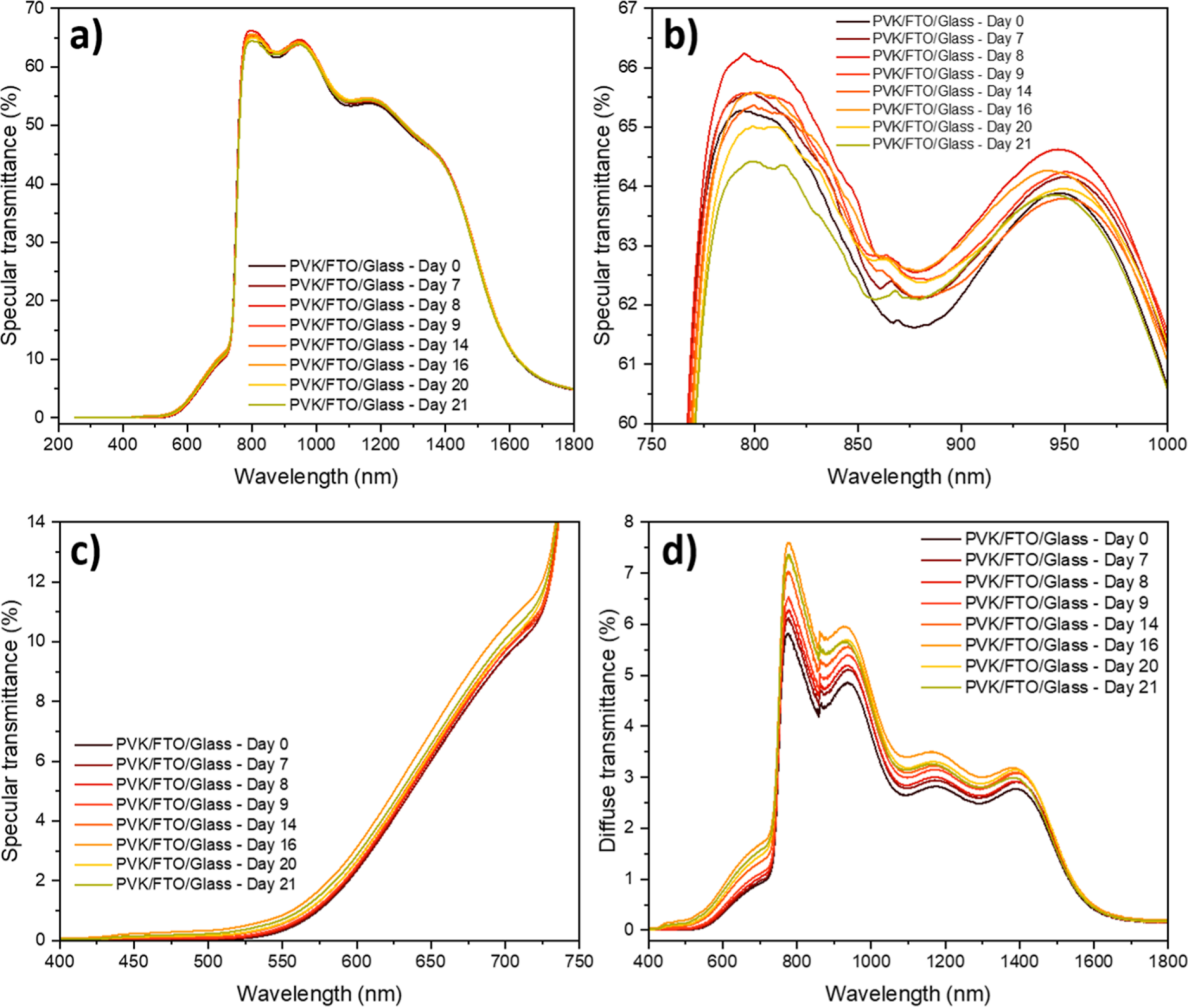
Specular and diffuse transmittance measurements over time by the PVK film side. (a) Represents the specular transmittance from 200 to 1800 nm, while (b) displays a zoom from 750 to 1000 nm (before absorption edge), (c) represents a zoom from 400 to 750 nm (after absorption edge), and (d) is the diffuse transmittance.

This pattern becomes clearer in the zoom presented in Figure 3b, focused on the 750–1000 nm region. The spectra do not evolve in a monotonic fashion. For example, at 800 nm, the transmittance increases from 65.27% (Day 0) to 66.22% (Day 8) but then declines to 64.40% by Day 21. These trends are consistent with an initial reduction in optical losses followed by the formation of degradation products or morphological changes that enhance scattering and partially offset the early optical gains. Any subtle spectral shifts remain within experimental dispersion but are compatible with a transition from a mixed perovskite phase toward PbI_2_-rich domains, commonly reported to form after a couple of days of aging.^24–26^

Figure 3c shows the specular transmittance in the visible range, where the pristine perovskite strongly absorbs. Even in this high-absorption region, a small but reproducible increase in the transmittance is observed with time, particularly between 450 and 650 nm. This indicates that the perovskite layer does not simply form an additional absorbing overlayer but gradually loses effective optical density as it degrades, either by thickness reduction or by partial phase transformation. The preservation of the spectral shape again suggests that the main effect of degradation is the attenuation of the absorption strength rather than the appearance of new absorption bands.

In contrast, the diffuse transmittance (Figure 3d) exhibits a more pronounced evolution with aging. The intensity of the scattered component increases markedly from Day 0 to Day 21 across the whole spectrum, with particularly strong changes around the broad features near 800–900 nm and in the near-infrared region. This behavior is consistent with degradation-induced microstructural damage: as the perovskite decomposes, it is expected to generate additional refractive-index inhomogeneities (such as PbI_2_ crystallites, voids, and roughened interfaces), which act as efficient scattering centers.^27–30^

As discussed in the literature, in spatially confined and disordered systems, absorption and scattering contributions become intrinsically coupled due to indirect optical transitions, making their strict deconvolution challenging. In this context, the observed optical evolution can be consistently interpreted through the combined analysis of specular and diffuse components.^31^

Taken together, the time-resolved measurements in Figure 3 illustrate that ambient exposure leads to a gradual degradation of the perovskite layer, manifested optically by a change in transmittance, even in the sub-bandgap region, and a substantial enhancement of the diffuse component.

Raman measurements were performed (Figure S1) to probe possible structural changes during aging. However, no clear or systematic spectral features associated with degradation products were observed, which can be attributed to the intrinsically weak Raman signal and low signal-to-noise ratio. As reported in the literature, the Raman response of pristine perovskite films is typically weak and can be easily obscured by noise, making it difficult to extract clear spectral features.^32–34^

When the measurements are performed from the glass side (glass/FTO/PVK configuration), the general degradation trends observed previously are preserved, but the spectral signatures become more moderate. In Figure 4a–c, the specular transmittance follows the same previous behavior (Figure 3a–c), but the magnitude of the increase is slightly smaller than in the PVK-illuminated geometry, which is consistent with the fact that glass-side illumination first encounters a smooth, nonscattering substrate before reaching the degrading film.

**Figure 4:**
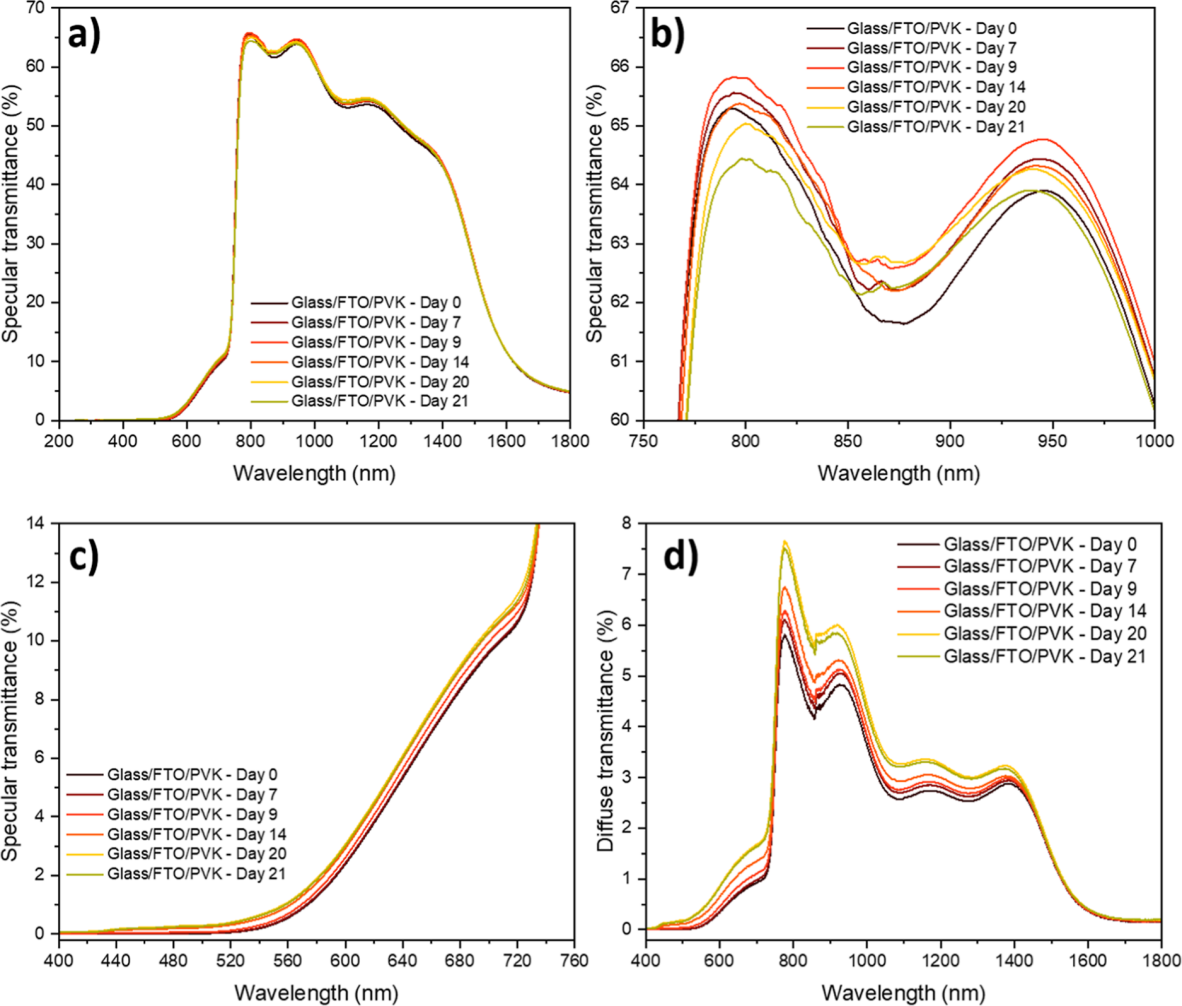
Over time measurements of specular and diffuse transmittance by the glass side. (a) Being the specular transmittance (200 to 1800 nm), (b) the zoom obtained before the absorption edge (750–1000 nm), (c) the zoom after the absorption edge (400–750 nm), and (d) the diffuse transmittance between 400 and 1800 nm.

In Figure 4d, the diffuse transmittance also increases with aging, although to a lesser extent compared to that of the PVK-side measurements. This can indicate that degradation still generates additional scattering centers, such as PbI_2_ crystallites, and index fluctuations, but the geometry partially suppresses the contribution of the rough PVK/FTO interface, reducing the apparent intensity of the diffuse scattering. Overall, the optical evolution measured from the glass side confirms the same degradation mechanism identified previously but with attenuated scattering effects due to the different illumination path.

When illumination is incident on the perovskite side, the total reflectance decreases progressively with aging (Figure 5a), but the trend is most pronounced at ∼900 nm below (Figure 5b). As the material degrades under ambient exposure, the perovskite layer becomes optically less reflective, probably due to increased absorption associated with defect-rich degradation products and structural disorder, which dampens the reflected light, or the enhanced scattering phenomenon becomes prominent.^35,36^

**Figure 5:**
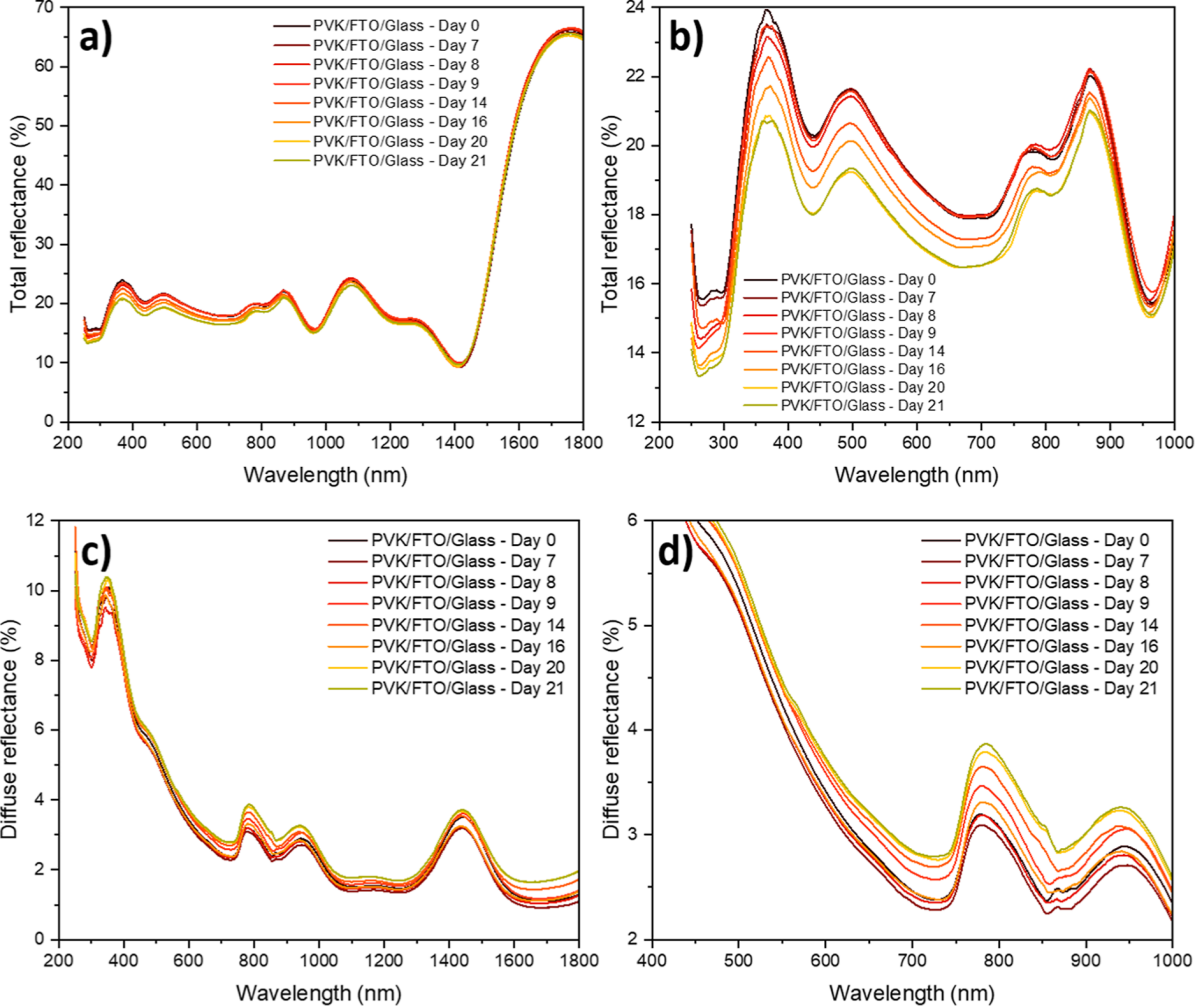
Total and diffuse reflectance measurements from the PVK illumination side. (a) Consists of the entire spectrum (200–1800) while (b) is the zoom between 200 and 1000 nm for total reflectance. The diffuse reflectance is illustrated in (c,d). (c) Being the entire spectrum again and (d) the zoom between 400 and 1000 nm.

In contrast, the diffuse reflectance exhibits a slight but systematic increase over time (Figure 5c,d). Again, this behavior can indicate the gradual formation of additional scattering centers, such as grain-boundary roughening, microcracks, and small PbI_2_ crystallites, resulting from morphological degradation.^24,25,27–30^ Although this scattered component increases, it remains comparatively small and is outweighed by the larger reduction in specular reflectance, leading to an overall decline in total reflected intensity. The combined trends suggest that aging drives both enhanced scattering and increased optical losses, with no evidence of stabilization within the first 21 days.

When illumination is incident from the glass side, the optical response follows the same overall degradation trend observed under PVK-side illumination but with a smaller magnitude of variation. Specifically, the total reflectance decreases with aging (Figure 6a,b), mirroring the behavior previously identified for perovskite-side measurements (Figure 5a,b). Nonetheless, the monotonic decrease confirms that absorption-driven degradation dominates in both illumination geometries. The diffuse reflectance also displays a slight but consistent increase over time (Figure 6c), in agreement with the trend identified for PVK-side illumination (Figure 5c,d).

**Figure 6:**
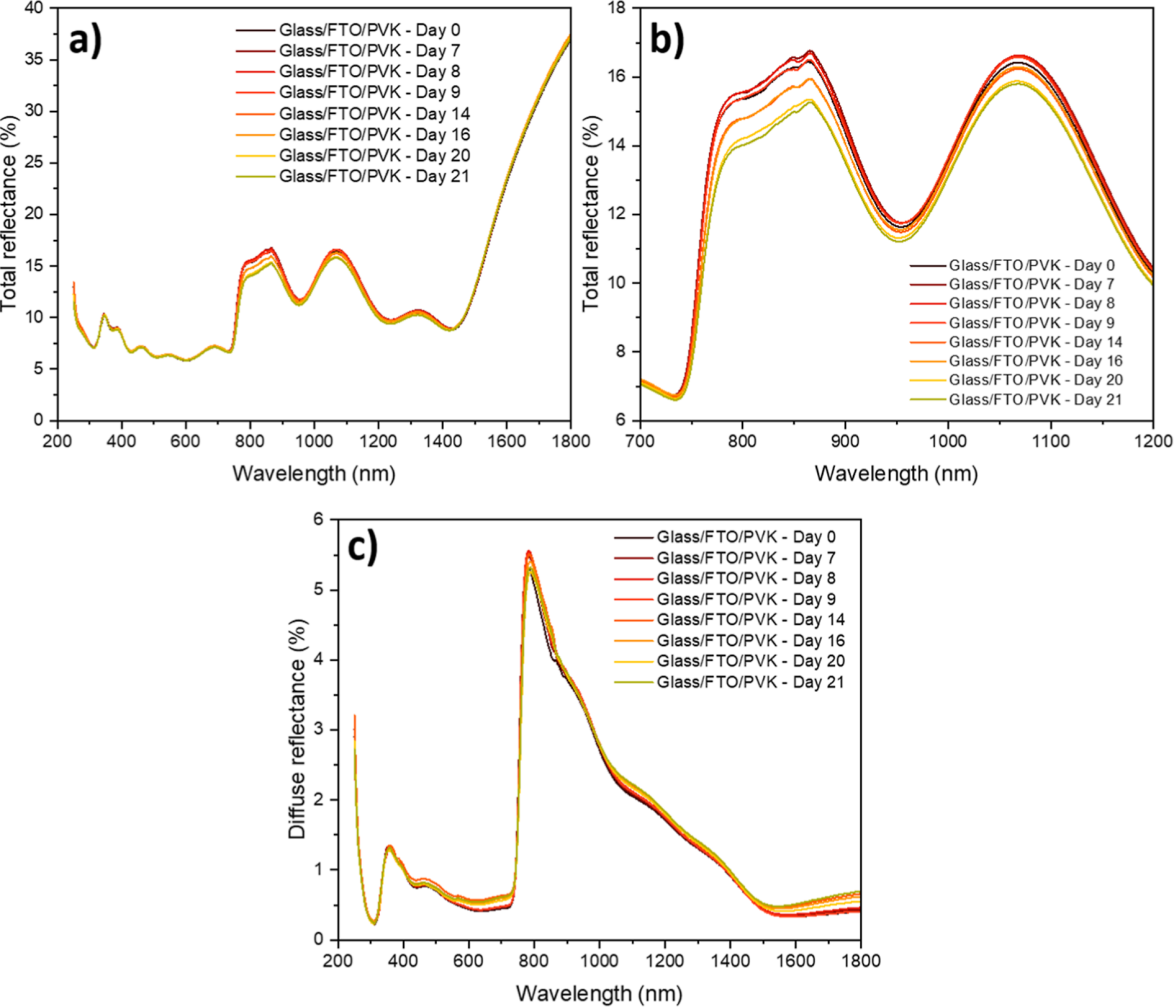
Total and diffuse measurements were performed on the glass side of the sample. (a) Is the total reflectance in the entire spectrum, (b) is the same spectrum but a zoom between 700 and 1200 was applied for better analysis, and (c) is the diffuse reflectance from 200 to 1800 nm.

Taken together, the reflectance measurements from both illumination geometries reveal a consistent optical signature of perovskite degradation under ambient exposure (Figures 5a,b and 6a,b). In both cases, perovskite-side and glass-side illumination, the total reflectance decreases monotonically over time, with the Day 0 spectra displaying the highest intensity and all subsequent measurements showing progressively lower values, probably due to the formation of defect-rich and chemically modified phases that introduce additional loss channels and suppress the amount of reflected light returned to the detector. The modulation is more pronounced when the incident beam first interacts with the PVK/FTO interface, but the direction of illumination affects only the magnitude and not the direction of the optical evolution.

The combined analysis of the specular and diffuse transmittance and reflectance measurements indicates that the scattering observed during aging cannot be attributed to a single interface. The bare glass/FTO substrate already exhibits significant diffuse reflectance (Figure 2d), demonstrating that the FTO layer is intrinsically a strong source of scattering due to its granular morphology and surface roughness.^37,38^ When the perovskite film is deposited, the diffuse component between 400 and 800 nm decreases, revealing that the PVK/air interface is optically smoother than the exposed FTO surface. Therefore, the external PVK/air interface is not the dominant source of scattering in the pristine state.

In summary, the combined transmittance and reflectance results show that the optical evolution of the perovskite film during aging arises from the interplay between absorption, scattering, and the intrinsic asymmetry of the glass/FTO/PVK heterostructure. The differences observed between illumination from the PVK side and from the glass/FTO side demonstrate that light–matter interaction strongly depends on the optical pathway, with the FTO layer acting as a primary source of scattering while the perovskite film initially smooths this interface and later contributes additional disorder as it degrades. Over time, the progressive decrease in specular transmittance and total reflectance, together with the systematic increase in diffuse components, indicates the formation of morphological irregularities, local refractive-index fluctuations, and possible PbI_2_-rich domains that enhance optical losses and modify the balance between scattering and absorption. Nevertheless, the preservation of the absorption edge probably shows that the fundamental bandgap remains essentially unchanged within the studied period. Overall, the optical degradation is governed mainly by moderate increases in disorder and additional absorption pathways, establishing the physical scenario necessary for the analysis of the derived optical quantities that follows.

Figure 7a presents the temporal evolution of the absorption coefficient α obtained from the combined transmittance and reflectance measurements.^39^ Overall, the curves remain similar in shape throughout the period, but a gradual reduction in α is observed at higher photon energies. In the region above approximately 2.2 eV, the maximum values of α decrease with aging, indicating a moderate reduction in the film’s overall absorption at shorter wavelengths. In the vicinity of the absorption edge (1.55–1.75 eV), the position and general profile of the transition remain nearly unchanged, suggesting that the fundamental bandgap does not shift appreciably over the measured time interval. The curves also converge in the sub-bandgap region below ∼1.4 eV, indicating no evidence of new low-energy absorption features associated with midgap states or additional optical transitions. These observations point to a progressive, but relatively modest, decrease in absorption at higher energies as the material ages, without clear spectral signatures that would allow definitive identification of specific degradation phases or products. The optical data therefore support a scenario in which the perovskite film undergoes changes that lower its absorption efficiency at higher photon energies, while its band edge behavior and sub-bandgap absorption remain essentially stable over the period.

**Figure 7:**
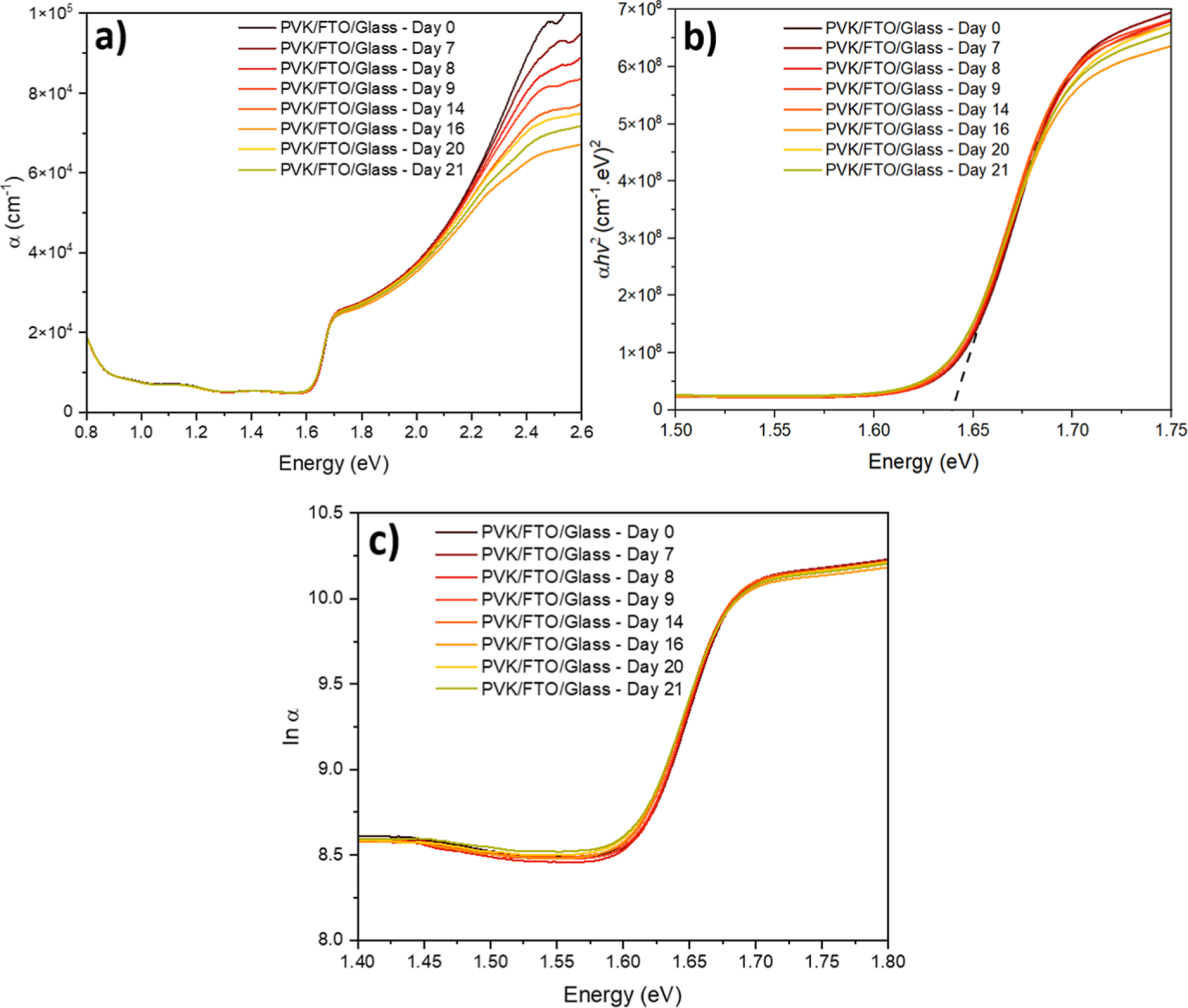
calculated absorption coefficients are displayed in (a) while the Tauc plot extrapolation to acquire the bandgap values is illustrated in (b) and the ln of the absorption coefficient to get the Urbach energy is shown in (c).

In Figure 7b, the representation of α*hv* ^2^ versus photon energy is used to visualize the direct-transition region and to estimate the optical bandgap. Over the period, the overall shape of the curves remains similar, and only minor changes are observed near the absorption edge. The extrapolated intercept obtained from the linear region shows little to no measurable shift, staying within the expected uncertainty associated with the choice of the fitting interval. Based on this, there is no clear experimental evidence of a significant change in the bandgap during aging. The slight reduction in slope near the edge is consistent with the modest decrease in absorption amplitude noted previously, but this effect does not allow a definitive conclusion about changes in electronic structure.

Figure 7c shows the ln α representation commonly used to examine the Urbach region near the absorption edge. Over the 21 days, the curves remain largely similar in shape, with only subtle changes observable in the tail region. A slight reduction in steepness can be noted, but the variation is modest and not strongly pronounced, indicating that any evolution in the absorption tail is relatively small within the experimental sensitivity.

Importantly, no clear emergence of new sub-bandgap absorption features is observed throughout the aging period. This suggests that, within the resolution of steady-state optical absorption measurements, there is no evidence of optically dominant midgap states or additional electronic transitions in the forbidden region. However, the absence of distinct spectral signatures in this region does not strictly rule out the formation of midgap states. Such states, if present, may exist at low densities, be spatially localized, or exhibit weak optical cross sections, making them difficult to detect using absorption-based techniques alone. More sensitive methods, such as nonthermal anti-Stokes photoluminescence, could, in principle, probe these states with higher sensitivity.^40^

Within this time frame, there is no strong optical evidence for the emergence of new sub-bandgap features or for a substantial change in the density of tail states, and the intrinsic band-edge position appears essentially unchanged.

In the last derivative parameter, the haze analysis presented in Figure 8 provides a complementary view of how the balance between specular and diffuse optical components evolves with aging. In both transmission and reflection geometries, the haze curves for all days remain closely grouped, exhibiting only small-wavelength-dependent fluctuations and no systematic monotonic trend. This behavior indicates that although degradation processes clearly increase the diffuse components observed in the raw transmittance and reflectance spectra, the relative contribution of scattered light to the total transmitted or reflected signal does not undergo a substantial transformation over the measured period.

**Figure 8:**
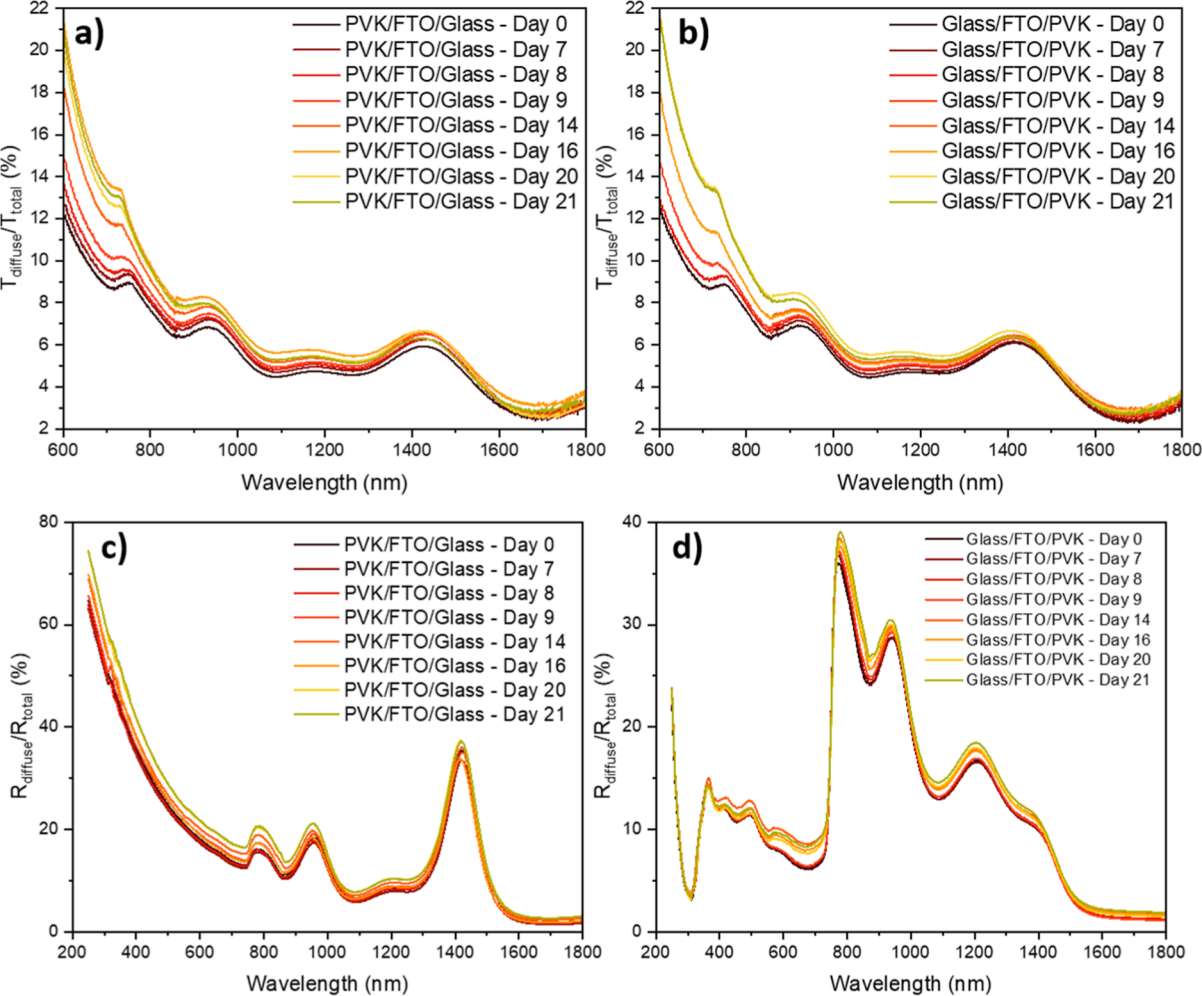
Haze measurements. (a) Is the transmittance illuminated by the PVK side, while (b) is by the glass side. (c) Is the reflectance by the PVK side and (d) is by the glass side.

In the transmission configuration (Figure 8a,b), haze values change only slightly with time, and the differences between PVK-side and glass-side illumination remain modest. This confirms that the generation of new scattering centers, such as PbI_2_ crystallites, or local index inhomogeneities is too limited to produce a strong shift in the diffuse-to-specular ratio. The smoother response from the glass-side illumination further reinforces the conclusion that the FTO/PVK interface rather than the PVK/air surface plays the dominant role in determining the scattering behavior.

A similar picture is observed in the reflection haze (Figure 8c,d), where only minor variations appear throughout the period. Although diffuse reflectance measurements showed a slight increase over time, the overall haze remained nearly constant, indicating that the increase in scattered light was partially compensated by the concurrent loss of specular reflectance due to enhanced absorption and disorder. As a result, haze does not amplify significantly, even as degradation progresses.

Overall, the haze results demonstrate that the optical aging of the Cs_17_/Br_17_ film is governed more strongly by absorption-driven attenuation and moderate scattering increases rather than by the emergence of highly efficient scattering centers. Thus, haze evolution appears to be a secondary indicator of degradation, consistent with the trends observed in the transmittance and reflectance measurements.

## Conclusion

This work investigated the optical evolution of Cs_17_/Br_17_ perovskite thin films under ambient aging by using combined specular and diffuse transmittance and reflectance measurements. The results show that degradation does not follow a simple monotonic behavior across all of the optical parameters. In particular, specular transmittance exhibits a nonmonotonic evolution, with an initial increase followed by a gradual decrease, while diffuse transmittance increases systematically, indicating the progressive formation of scattering centers associated with structural disorder.

In parallel, total reflectance decreases with aging, whereas diffuse reflectance shows a slight increase, revealing that degradation involves the combined effects of enhanced scattering- and absorption-related optical losses. These trends demonstrate that the optical response arises from the interplay between structural disorder, scattering, and absorption processes. Despite these changes, the absorption edge, optical bandgap, and Urbach tail remain essentially unchanged, indicating that the fundamental electronic structure of the perovskite is largely preserved within the investigated period.

The comparison between both illumination geometries further shows that the FTO substrate acts as an intrinsic source of scattering, while the perovskite layer initially reduces the optical contrast and progressively introduces additional disorder as degradation proceeds.

Overall, the combined analysis of specular and diffuse optical components provides a more comprehensive description of degradation than that of conventional absorption-based approaches. This methodology offers a practical and nondestructive framework for monitoring stability in perovskite thin films and can support the development of more durable optoelectronic devices. Future studies should extend this approach to complete device architectures, enabling the investigation of degradation mechanisms across all functional layers and their interfaces under realistic operating conditions.

## Methods

### Materials and Sample Preparation

Commercial FTOs (MSE supplies), 7–8 Ohm/sq (TEC 7) of resistance, were used as substrates. They were cleaned by ultrasonic baths for 50 min. Ten minutes at each compound, following the next steps: neutral detergent (dextran) with deionized water (1:10), pure deionized water, acetone (99.9%, Synth), and isopropanol (99.9%, Synth).

The perovskite films were deposited on the cleaned substrates by the spin-coating deposition method. The molar ratio of 17:83, CsPbBr_3_/FAPbI_3_, respectively, was used by applying the following precursor solution (all chemicals were purchased and used without further purification): FABr (1.2 mmol–99.99%, Greatcell), PbBr_2_ (1.2 mmol–99.998%, Sigma-Aldrich), PbI_2_ (1.2 mmol–99.999%, TCI Chemicals), FAI (1 mmol–99.99%, Greatcell), and CsI (0.2 mmol–99.999%, Sigma-Aldrich), in anhydrous *N*,*N*-Dimethylformamide:Dimethyl sulfoxide–DMF (99.8%, Sigma-Aldrich):DMSO (>99.9%, Sigma-Aldrich) (4:1 v/v). The resulting solution was left at 70 °C stirring overnight and then filtered using a 0.45 μm syringe filter. The final solution was spin-coated at 1000 rpm for 10 s and then at 6000 rpm for 25 s. After 20 s, 200 μL of anhydrous chlorobenzene (99.8%, Sigma-Aldrich) was added as an antisolvent. After the aforementioned procedure, the film was annealed at 120 °C for 30 min on a hot plate to evaporate the solvent and improve crystallization.

The perovskite sample under analysis (abbreviated as PVK) consists of a glass/FTO/PVK heterostructure, and a schematic representation of the sample is illustrated in Figure 9. Moreover, all samples were characterized with the incident beam directed from both sides, glass/FTO/PVK, and PVK/FTO/glass, to provide a complementary assessment of the optical response under different illumination geometries.

**Figure 9:**
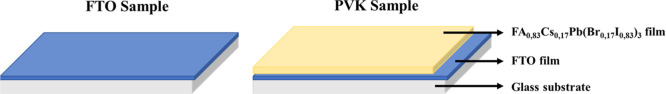
Schematic representation of the FTO substrate on the left side, composed of glass and FTO (glass/FTO), and the perovskite sample deposited by spin-coating (glass/FTO/PVK) on the right side.

### Characterization Technique

Optical measurements were performed using a PerkinElmer UV/vis/NIR Lambda 1050 spectrometer equipped with a Labsphere 150 integrating sphere, allowing us to measure both the transmittance and reflectance, in the specular and diffuse or the total mode for reflectance measurements. In optical measurements, the transmitted light can either propagate through the sample along the original path (normal incidence) or be scattered in multiple directions. The component that emerges at the same angle as the incident beam is termed specular transmittance, while the component redistributed over a range of angles is known as diffuse transmittance, and the total transmittance corresponds to the sum of both contributions. A similar distinction applies to reflectance. In this work, we report specular and diffuse transmittance as well as total reflectance (specular + diffuse) and diffuse reflectance. The separation of these components is essential to distinguishing between direct transmission and scattering effects, which play a key role in the degradation of perovskite films and cannot be resolved from total transmittance or reflectance alone.

The measurement configuration is defined by the incident beam direction and the integrating sphere geometry, which enables the separation of specular and diffuse components. Measurements were performed with the incident beam directed from both sides of the sample (PVK/FTO/glass and glass/FTO/PVK), allowing a comparative assessment of the optical response under different illumination geometries. All spectra were acquired in the 250–1800 nm wavelength range.

The Raman spectra were acquired using a LabRAM Odyssey Raman spectrometer equipped with an excitation laser at 532 nm. Spectral acquisition was performed in the 50–200 cm^–1^ range.

### Optical Characterization

The optical characterization was conducted through sequential transmittance and reflectance measurements. First, specular transmittance was recorded with the incident beam directed onto the perovskite face of the sample (PVK/FTO/glass). Next, diffuse transmittance was measured under the same geometry. The sample was then rotated 180° relative to an axis tangent to its surface, exposing the glass face to the incident beam (glass/FTO/PVK), and the same pair of measurements (specular and diffuse transmittance) was repeated. Following the transmittance acquisition, the sample was positioned for reflectance measurements. In this configuration, total reflectance was measured first, followed by diffuse reflectance, using the same sample orientation order relative to the incident beam as described above. Throughout the entire procedure, the samples were stored under an ambient atmosphere and kept in the dark, avoiding direct light exposure. The average temperature and relative humidity during the aging period were 27.6 °C and 53.9%, respectively.

### Measurement Procedure

The refractive index was obtained from the interference fringes observed in the transmittance spectra within the transparent region, using the method proposed by Cisneros.^41^ Since this method was formulated for a single film deposited on a substrate, an approximation was employed in which the glass + FTO layers were treated as an effective substrate. Using the measured transmittance and reflectance in the high absorption region, away from the interference fringes, the absorption coefficient (α) for the films was estimated using eq 1 ^39^

1 $$\alpha =\frac{1}{d}\ln\left[\frac{{\left(1-R\right)}^{2}}{2T}-\sqrt{\frac{{\left(1-R\right)}^{4}}{2T^{2}}+R^{2}}\right]$$

where α is the absorption coefficient, *d* is the film thickness, *T* is the transmittance, *R* is the reflectance, and *A* is a proportionality constant (typically taken as 1).

From the calculated absorption coefficient values, the optical bandgap (*E*_g_) was estimated using the Tauc^42^ relation using eq 2:

2 $${\left(\alpha hv\right)}^{r}=B\left(hv-E_{g}\right)$$

where *B* is a proportionality constant and *r* depends on the nature of the optical transition. In the present work, *r* = 2 was adopted, corresponding to an allowed direct transition, since lead halide perovskites are commonly described as direct-bandgap semiconductors.^43^ Accordingly, *E*_g_ was estimated from the linear extrapolation of the (α*hv*)^2^ versus *hv* plot to (α*hv*)^2^ = 0.

For photon energies below the bandgap, absorption is dominated by Urbach tail states, described by the empirical relation in eq 3 ^44^

3 $$\ln\alpha =\ln\alpha_{0}+\left(\frac{hv-E_{1}}{E_{0}}\right)$$

Where *E*_1_ is the Urbach focus, α is a constant, and *E*_0_ is the Urbach energy, a parameter directly related to the degree of structural disorder and the density of localized states within the forbidden region.^44^

Lastly, haze was evaluated as the ratio between diffuse and total transmittance, providing a quantitative measure of light scattering within the films. Higher haze values indicate increased light scattering due to structural disorder and inhomogeneities. The haze was calculated observing the following eqs 4 and 5, where Haze_T_ is the transmittance Haze, Haze_R_ is the reflectance Haze, *T*_diff_ is the diffuse transmittance, *T*_total_ is the total transmittance, *R*_diff_ is the diffuse reflectance, and *R*_total_ is the total reflectance^45,46^

4 $${Haze}_{T}=\frac{T_{diff}}{T_{total}}$$

5 $${Haze}_{R}=\frac{R_{diff}}{R_{total}}$$

## Supplementary Material



## Notes

Raman spectra of aged samples (PDF)
