# Supplementary material for: Optical Scattering Evolution during Ambient Aging of Cs/FA Alloyed Perovskite Thin Films
Source: ACS Omega. 2026 May 30;11(23):34274–86. doi: 10.1021/acsomega.6c01763 (PMC13280884; doi:10.1021/acsomega.6c01763)
Supplement: Supplementary file 1 [file ao6c01763_si_001.pdf]

Supporting information

Optical Scattering Evolution during Ambient Aging of Cs/FA Alloyed Perovskite Thin Films

Lucas Caniati Escaliante[a*], Lucas Jorge Affonço[b,c], Stevan Brayan Oliveira dos Santos[a], Larissa de Oliveira Garcia[d], Silvia Leticia Fernandes[c], André Luis de Jesus Pereira[e], Carlos Frederico de Oliveira Graeff[a,b], José Humberto Dias da Silva[a,b]

[a] *School of Sciences, Graduate Program in Materials Science and Technology – POSMAT, Universidade Estadual Paulista – UNESP, Bauru, São Paulo, 17033-360, Brazil.*

[b] *School of Sciences, Physics and Meteorology Department, Universidade Estadual Paulista – UNESP, Bauru, São Paulo, 17033-360, Brazil*

[c] *ONINN Centro de Inovação, Belo Horizonte, Minas Gerais, 31035-536, Brazil*

[d] *Faculty of Physical Engineering/Computer Sciences, University of Applied Sciences Zwickau, Zwickau, 08056, Germany*

[e] *Plasma and Processes Laboratory, Division of Fundamental Sciences, Instituto de Tecnologia Aeronáutica – ITA, São José dos Campos, São Paulo, 12228-900, Brazil*

** Email: lucas.caniati@unesp.br*

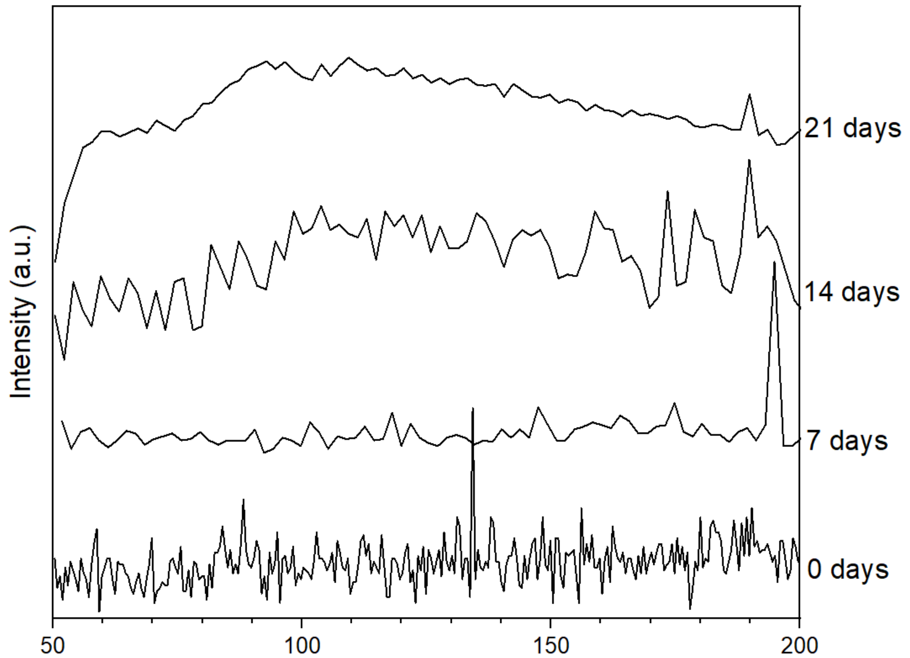

Figure S1. Raman spectra of the perovskite thin films at different aging times (0–21 days).
